# Depression as an early symptom and risk factor of dementia - a narrative review

**DOI:** 10.3389/fpsyt.2026.1786179

**Published:** 2026-04-10

**Authors:** Agnieszka Permoda-Pachuta, Piotr Obszanski, Zbigniew Grad, Małgorzata Futyma-Jędrzejewska, Piotr Ziemecki, Monika Dominiak

**Affiliations:** 1 Department of Neuroses, Personality Disorders and Eating Disorders, Institute of Psychiatry and Neurology (IPiN), Warsaw, Poland; 2 Department of Radiology, Institute of Psychiatry and Neurology (IPiN), Warsaw, Poland; 3 Department of Psychiatry, Psychotherapy and Early Intervention, Medical University of Lublin, Lublin, Poland; 4 Institute of Psychiatry and Neurology (IPiN), Warsaw, Poland

**Keywords:** Alzheimer’s disease, dementia, depression, differential diagnosis, late-life depression, risk factors

## Abstract

Depression is a common psychiatric disorder, while dementia represents a growing global health challenge, particularly in aging populations. Although substantial progress has been made in pharmacotherapy, neurodegenerative processes can only be partially slowed, and disease progression cannot be completely halted. Neurodegenerative diseases therefore remain largely incurable, underscoring the importance of early recognition and intervention. This raises an important clinical and conceptual question: does depression represent an early manifestation of dementia, act as a risk factor for its development or both? Understanding these relationships is essential for accurate diagnosis, appropriate treatment, and timely implementation of preventive strategies. This article presents a narrative review of the literature examining the complex relationship between depression and dementia, with a focus on clinical presentation, diagnostic challenges, and neurobiological mechanisms. Neuroimaging techniques such as MRI and PET, and in selected contexts SPECT, support the differential diagnosis of depression and dementia, although limitations in sensitivity and specificity persist. Inflammation has been extensively investigated as a shared pathological mechanism underlying both conditions. Emerging evidence also suggests that anti-amyloid therapies may be associated with improvements in depressive symptoms in selected patient populations, further highlighting overlapping pathophysiological pathways between depression and dementia. Improved understanding of the interplay between depression and dementia may facilitate earlier diagnosis, reduce diagnostic uncertainty, and support the development of more effective preventive and therapeutic strategies.

## 1 Introduction

The number of individuals afflicted with neurodegenerative diseases has been gradually rising in recent decades. This phenomenon is chiefly associated with the aging of the population and represents a significant clinical and social challenge, particularly in developed countries, especially in the context of dementia. Late-life depression is usually defined as the emergence of major depression in patients aged 60 and older. It is a significant health burden responsible for the increased number of completed suicides in this age group. Many symptoms of Major Depressive Disorder (MDD) as defined by DSM-5 and mild cognitive impairment as well as Major Neurocognitive Disorder (MND) overlap making differential diagnosis difficult. Projections indicate that by the year 2050, the number of individuals diagnosed with Alzheimer’s Disease (AD) and vascular dementia (VD), which currently represent the most prevalent forms of dementia, will reach approximately 135.5 million (1). It is therefore important to develop reliable diagnostic procedures in order to facilitate the treatment process. Differential diagnosis of depression and dementia in older individuals can be improved by the judicious weighing of risk factors associated with both conditions.

Researchers are working on new treatment options for dementia, but the disease is still considered incurable. Pharmacotherapy may partially inhibit neurodegenerative processes, although complete inhibition is not currently achievable. Results are nevertheless hampered by the relatively late initiation of treatment, as patients often consult a specialist only after a prolonged period of symptomatic cognitive impairment. The severity of pathophysiological changes in the brain in most cases prevents the symptoms from resolving despite treatment (2). The neurological determinations of developing AD are often indiscernible and do not appear until the later stages of the disease, with the result that patients are diagnosed too late (3–5).

The literature also reveals that reducing 10% of risk factors can reduce the onset of the disease in more than a million individuals (6). It is therefore important to look for prodromal symptoms and risk factors according to the common statement: prevention is better than cure. This leads to the question: Is depression a symptom of dementia, or does dementia occur in depression? Depression may represent an early manifestation of dementia, preceding the onset of overt cognitive decline. Prolonged, untreated depression is also considered an important risk factor for the later development of dementia. In addition, depressive disorders and early-stage dementia may coexist, which can complicate diagnosis and require careful assessment. From a clinical perspective, distinguishing between depression-related cognitive impairment and early dementia remains critical for appropriate treatment planning and prognostic evaluation. Therefore, the aim of this paper was to provide a narrative synthesis of the evidence on the relationship between depression and dementia and to highlight its relevance for neuropsychiatric diagnosis and treatment.

## 2 Methods

The present work is a narrative review based on a qualitative synthesis of the literature. Literature review was carried out using electronic databases: PubMed, Google Scholar. The following keywords were used: Alzheimer’s disease; dementia; depression; risk factors. Inclusion criteria included: only original papers, meta-analyses and review papers, research published from 2010-2025 with particular emphasis on the last 5 years and original publications in English. Exclusion criteria included: studies prior to 2010, studies published in a language other than English. The focus was on data on depression as an early sign of dementia, less on depression leading to dementia, and ignoring depression coexisting with dementia, as topics beyond the scope of this review. For the purposes of this review, authors decided not to separate dementia into individual types.

## 3 Depression and dementia

Late-life depression (LLD) can result in cognitive decline (depressive pseudo-dementia). At the same time depressive symptoms may be the first clinical sign that point to the development of dementia in the future (7).

LLD appears to be associated with a significantly higher risk for incident all-cause dementia, Alzheimer’s disease and vascular dementia. The risk of vascular dementia is significantly higher than the risk of Alzheimer’s disease in older adults with late-life depression according to one meta-analysis of population-based prospective cohort studies (8).

Depression in the elderly can present with typical symptoms: depressed mood, apathy, anhedonia and disturbance of diurnal rhythms, but in most cases cognitive impairment appears to dominate the clinical picture (up to 50% of patients). It has also been shown that cognitive impairment is greatest at the onset of a depressive episode and lasts longer in patients with late-life depression.

LLD is associated with high treatment resistance, frequent relapses, and cognitive impairment which persist despite proper and effective treatment. The previous history of depressive episodes, their length, number and severity play a major role. It is also known that comorbidities especially from the vascular incidents worsen the prognosis. However, the age of the first depressive episode plays the most significant role in the development of LLD.

Studies on the relationship between depression and dementia focus on three main areas. First, they examine depression as a potential risk factor for developing dementia. Second, they consider whether depression can be conceived as a prodromal syndrome of dementia. Third, they analyze how these two diseases co-occur in the same patient (9). Some patients with mild cognitive impairment will not develop dementia and return to normal cognitive status after treatment or even without implementing therapy. However, some of these individuals have a co-occurrence of depressive symptoms-the percentage varies between 27-37% and this may increase the risk of developing dementia in the future (10, 11). Taking into account the available research in differentiating depression as an independent risk factor for dementia from other confounding variables, the use of neuroimaging and biomarkers should be considered, with particular emphasis on amyloid PET scans or tau protein levels.

Moreover, numerous studies confirm that both conditions share common risk factors, and for dementia, these include low levels of education, head injuries, physical inactivity, smoking, excessive alcohol consumption, hypertension, obesity, and diabetes (12). It has been confirmed that the presence of a depressive episode and diabetes significantly increases the risk of developing dementia as does confirmation of the presence of Aβ- amyloid, pathological tau protein or alleles of apolipoprotein E (13–15).

It should also be noted that cognitive reserve has a protective function as does greater thickness of the cerebral cortex or a high level of education (16–18). In contrast, the development of LLD is associated with an inflammatory process that leads to damage of the brain vessels, amyloid deposition, damage and atrophy of the hippocampus, and reduced volume of the basal ganglia of the prefrontal region (19).

Depressive symptoms (dimensional diagnosis and symptom patterns) as well as depression as a categorical diagnosis (major depression) have been studied as potential risk factors of the onset of dementia in the future. It appears that only a categorical diagnosis of a major depressive episode raises the risk of developing dementia (20). Several studies on large populations also confirmed that the onset of major depressive disorder in middle age increased the risk of developing dementia (21, 22). However, the analyzed studies lack details concerning the specific features of depression such as the variability of depressive symptoms over time, as well as the course whether it consisted of multiple episodes, length of remission periods.

Environmental factors, including social isolation and a healthy lifestyle, have a significant impact on the development of depression and dementia. These factors may be intermediaries or modulators of depression, and thus affect the risk of developing dementia (23).

## 4 Inflammatory system and role of microglia activation

The relationship between the dysregulation of inflammatory and immune processes is also being investigated as contributing to the process of neurodegeneration in dementia (24).

Researchers are focusing on the link between the release of pro-inflammatory cytokines from macrophages or related cells, particularly interleukin IL-1β, IL-6 and tumor necrosis factor TNF-α and the emergence of neurovegetative symptoms of depression. Interestingly, the same processes that lead to stroke and other cardiovascular diseases also increase the risk of developing dementia. New hypotheses are also emerging regarding: hyperactivation of neuroinflammatory microglia and inflammation stemming from dysregulated gut-brain axis (25). All of these processes can increase the permeability of the blood-brain barrier and facilitate access of cytokines to the brain, contributing to depression and neurodegenerative diseases.

It has been shown that what follows is an increased local secretion of pro-inflammatory cytokines, chemokines (chemoattractant cytokines) and growth factors, including IL-6, IL-8/C-X-C motif chemokine ligand 8, monocyte chemoattractant protein-1 (MCP1/CCL2) and vascular endothelial growth factor (VEGF) (26, 27). This process induces bacterial translocation from the gastrointestinal tract and the activation of cytokine- producing inflammasomes in circulating myeloid cells and brain-resident glia (28, 29).

This leads to an increasing concentration of free radicals, weakening of the action of neurotrophins and consequently damage and loss of neurons and glia, which is the basic mechanism leading to the deterioration of cognitive functions and, in the later stages, dementia.

Understanding the pathophysiological processes allows us, (at this point only hypothetically) to use cytokine biomarkers to determine which of them (or rather their combinations) are responsible for maintaining the inflammatory process and thus related to the so-called inflammatory depression and dementia.

Additionally, it enables the use of targeted treatment with anti-inflammatory drugs or cytokine inhibitors together with standard antidepressants, which may consequently prevent dementia in selected patient populations (30).

Late-life depression can lead to neurodegeneration through inflammatory mechanisms, in which pro-inflammatory cytokines, including IL-6 and TNF-α, play an extremely important role in microglial activation, neuronal damage, and hippocampal atrophy (31–35).

Structural damage to the brain, especially in the hippocampus, may be an early indicator of neurodegeneration, not directly related to dementia, but predisposing to its development in the future. A detailed discussion of the role of proinflammatory cytokines in the pathogenesis of depression and dementia is beyond the scope of this review.

Patients with APOE-E4 show a higher risk of hippocampal atrophy. In depression, which can also result in a decrease in the volume of the hippocampus, the occurrence of APOE-E4 may be associated with the severity of these changes and the acceleration of the neurodegenerative process (36).

## 5 Use of imaging studies to differentiate depressive disorders from dementia

In this review we focused on magnetic resonance imaging (MRI) and positron emission tomography imaging (PET).

Studies confirm that patients with LLD have more neuropathological changes, more structural brain abnormalities and changes in cognitive functioning before and after a depressive episode. However, it is still unclear whether the structural abnormalities are related to the etiology of LLD or rather a consequence of it (37).

Interestingly research based on Diffusion Tensor Imaging (DTI) analysis has shown that there is an overlap in anatomical structures that are affected in both LLD and in patients with Mild cognitive impairment due to Alzheimer’s Disease (MCI-AD). These structures are corpus callosum, anterior cingulate cortex, uncinate fasciculus, superior longitudinal fascicle, anterior thalamic radiation (38–44). This suggest that both entities could have the same pathological pattern and thus simultaneously raise the suspicion of LLD and MCI-AD diagnosis. It follows that DTI alone is not a specific tool for primary differential diagnosis in such situations but can be valuable retrospectively after improvement of patient’s condition, since part of changes can normalize in LLD and tend to worsen in MCI-AD (45).

Early diagnosis of dementia appears to be an important factor in the effectiveness of treatment. Imaging studies can distinguish between different types of dementia, as well as other diseases. However, it should be emphasized that the purpose of the imaging study is to confirm the diagnosis made on the basis of the clinical picture (46).

MRI is widely used in clinical practice and can be helpful in the exclusion of reversible causes of cognitive impairment but has relatively low sensitivity in the diagnosis of dementia subtypes, especially early on.

In AD, reduction of hippocampal volume is observed. Published in 2017 studies show a correlation of reduction of the left anterior hippocampus and bilateral posterior cingulate cortex. A limitation of these studies was the relatively small sample of patients studied (47). Hippocampal atrophy in depression is the result of chronic stress and neuroplasticity disorders. These changes are often reversible with the use of pharmacotherapy or psychotherapy while in dementia they are part of a neurodegenerative process that leads to irreversible brain damage. However, the analysis of tests should be cautious due to the fact that neuroimaging cannot always confirm cognitive impairment, especially of executive function, as evidenced by neuropsychological tests or the amount of amyloid deposited (48, 49).

There are differences in the atrophy of the hippocampus between LLD and MCI-AD. Global medial temporal atrophy is not associated with LLD and MCI-AD, however it is seen in major neurocognitive disorder due to AD. CA1 subregion and subiculum are the subregions which tend to be affected earlier in MCI-AD and serve as a potential marker of development of AD. The above mentioned subregions are not typically damaged in the course of LLD (50, 51).

In order to differentiate between late-life depression (pseudodementia) and AD, it is recommended to perform PET examination.

There are slight differences of sensitivity and specificity among radiotraces and those made for detection of beta-amyloid deposition (i.e. flobetapir 92% and 100% respectively (52)) and neurofibrillary tangles detectors (i.e. flortaucipir 92.3%-100%, and 52-92% respectively (53)) are most sensitive and specific followed by synaptic metabolism evaluators (fluorodeoxyglucose-PET 91% and 86% respectively (54). The latter combined with volumetric MRI seems to be the most accessible, whereas negative result of amyloid-PET virtually rules out AD but it is less attainable (55–57).

FDG-PET has high sensitivity and specificity for AD, while PET with ligands for amyloid and tau can improve the differential diagnosis of AD and non-AD dementias, including detection at the prodromal stage.

In Alzheimer’s disease, there is reduced metabolism of the hippocampal region, inferior parietal lobe, lateral temporal lobe and posterior cingulate cortex, and to a lesser extent the prefrontal cortex (58).

The consensus presented by the European Association of Nuclear Medicine and European Academy of Neurology includes conclusions that a regular FDG-PET scan provides strong evidence for depressive pseudo-dementia while the typical pattern of hypometabolism for one of the degenerative dementias argues against pseudo-dementia, although subtle frontal hypometabolism can occur in patients with mental disorders (59).

Currently, fMRI and PET scans can help distinguish depression in dementia from neurodegenerative dementia. Factors considered in the analysis are changes in brain activity, blood flow, metabolism and accumulation of amyloid and tau protein, changes in blood flow, and white matter integrity (60, 61).

Changes in blood flow in the brain as well as in white matter integrity caused by hypoxia and neuronal damage can contribute to the development of dementia.

Cerebral hypoperfusion, especially in the area of the hippocampus, prefrontal cortex and limbic systems, can cause a decrease in glucose and oxygen levels and disturbances in the balance of neurotransmitters (serotonin, dopamine, noradrenaline), which are often associated with symptoms of depression and cognitive decline. However, these findings have not been universally replicated (62, 63).

Single photon emission computed tomography (SPECT) can also be used in diagnosis with good results but it has lower resolution than PET (64). As PET imaging has become increasingly accessible, the routine use of SPECT appears more limited, although it may retain value in selected clinical and research contexts.

Magnetic resonance spectroscopy (MRS) is a promising tool and currently is used as a supplement in ambiguous cases but comes with limitations due to the technique and thus cannot be use widely. The N-acetyloasparaginase (NAA) reduction and myoinosytol (mI) increase has been noted in AD. As the changes in those metabolites increase in the course of the disease the NAA/mI ratio is a promising indicator of response to the pharmacological therapy (65).

## 6 Anti-amyloid treatment and depression

Some authors suggest that the implementation of anti-amyloid treatment may play an important role in the therapy of depression in the elderly (66).

To reduce the risk of dementia in people with depression at a late age, therapeutic approaches should be modified, including: pharmacotherapy, cognitive therapies. The focus should be on early intervention. Pharmacological treatment should include the use of: neuroprotective and anti-inflammatory drugs as well as antipsychotics (67, 68).

Monoclonal antibodies are being studied as potential agents reducing beta-amyloid in the brain, but patients with significant depressive symptoms have not been included in clinical trials, although it is known that depression is associated with dementia (69–71).

Reduction of depressive symptoms improves patients’ cooperation in the treatment process, and quality of life. It should also be mentioned that it reduces the risk of suicidal behavior in patients with dementia disorders (72, 73).

It should also be noted that treatment of late-life depression can be difficult, and often only many modifications of treatment bring tangible benefits in the form of resolution of symptoms. One in three patients responds satisfactorily to the first pharmacological treatment implemented, hence it is often the second or third therapy that brings the expected results.

In addition, psychotherapy is used in treatment, which is a challenge for patients with cognitive impairment.

It should also be taken into account that most of the studies involve people over the age of 65. However, it is known that the pathophysiological process in dementia can develop much earlier, before the clinical manifestations of dementia become apparent. Therefore, special attention must be paid to the fact that episodes of depression, understood as a factor in the development of dementia in the future, may affect younger people. And in connection with the increase with age of vascular diseases contribute to the development of dementia. One of the few studies conducted on a large population of people over 50 confirmed that a depressive episode is a factor in the development of dementia (74).

In a recent study focusing on the pathophysiological mechanisms regulating the depression- dementia relationship Huang et al. have shown that these depend on genetic predispositions, immune system dysfunction, but also on the accumulation of biomarkers associated with Alzheimer’s disease (e.g. amyloid-β and tau) and abnormalities in the brain structure (75).

It is also known that damage to the prefrontal cortex correlates with depressive behavior, especially when there is a reduction in the volume of the hippocampus but also damage to the gray matter through a reduction of dendritic arborization in the process of neurogenesis (76). Moreover, in depressive disorders, neuronal death can occur as a result of increased neurodegenerative changes and through activation of inflammatory pathways and cardiovascular mechanisms that increase the risk of stroke and small vessel disease leading to white matter lesions (77, 78).

Lesions of the central nervous systems visible as white matter hyperintensity in patients with late- age depression were found to be related to disorders of the cortical and subcortical gray matter, which was involved in cognitive control, sensorimotor processing, and affected the speed of processing and attention (79).

Early intervention for depression, especially with cognitive therapies and effective drug treatment, can reduce cognitive impairment and thus the risk of dementia.

## 7 Conclusions

The relationship between depression and dementia is complex and multidirectional. Depression may represent an early manifestation of an underlying neurodegenerative process, act as a risk factor for the later development of dementia, or coexist with early-stage dementia, thereby complicating clinical assessment. Distinguishing between these scenarios remains a significant diagnostic challenge, particularly in older adults presenting with affective and cognitive symptoms.

Current evidence indicates that although pharmacotherapy may partially inhibit neurodegenerative processes, disease progression cannot be fully halted, highlighting the importance of early recognition and timely intervention. Accurate identification of depressive symptoms in the context of cognitive decline is therefore essential for appropriate treatment planning and prognostic evaluation.

Most studies have shown that depression may be a risk factor for dementia as well as a prodromal syndrome of developing dementia and age is a prognostic factor that worsens prognosis (9). Research seems to confirm that depression increases the risk of developing any type of dementia (80, 81).

Meta-analyses have not provided a clear answer, but it is known that depression was listed among the potentially modifiable factors in the development of dementia, according to data from the Lancet Commission on Dementia’s 2024 report (82). Other potentially modifiable factors include vision loss and high cholesterol, low education levels, head injuries, physical inactivity, smoking, excessive alcohol consumption, hypertension, obesity, diabetes, hearing loss, infrequent social contact and air pollution.

Imaging techniques such as MRI, especially fMRI and PET are utilized in the differential diagnosis of depression and dementia however there is still room for improvement in the area of specificity and sensitivity.

The many links between depression and dementia still demand the light of further research to be fruitfully employed in the service of diagnosis and therapy.

Improved understanding of the interplay between depression and dementia may facilitate earlier diagnosis, reduce diagnostic uncertainty, and support the development of more effective preventive and therapeutic strategies. Longitudinal clinical observation and the integration of neuroimaging and biological markers remain crucial for clarifying causal relationships and guiding future research.
